# Spontaneous Retroperitoneal Methicillin-Resistant Staphylococcus aureus Abscess in a Pediatric Patient

**DOI:** 10.7759/cureus.16111

**Published:** 2021-07-02

**Authors:** Biruk Almaz, Rosmeld Castillo, Christopher Nemeh, Christopher Gitzelmann

**Affiliations:** 1 Surgery, Rutgers University, Newark, USA; 2 Pediatric Surgery, Rutgers University, Newark, USA

**Keywords:** pediatric surgery, retroperitoneal abscess, abscess, spontaneous abscess, pediatric, pediatric abscess, retroperitoneum abscess, case report pediatric, mrsa abscess, methicillin resistant staphylococcus aureus

## Abstract

Retroperitoneal abscesses are relatively uncommon in infants and children. They carry a high rate of morbidity due to insidious onset and pose a diagnostic challenge. Here we report a case of spontaneous retroperitoneal methicillin-resistant *Staphylococcus aureus* (MRSA) infection in a two-year-old patient. The patient was successfully treated with antibiotics and surgical washout and drainage. A retroperitoneal abscess is usually found in patients with a history of osteomyelitis, seeding of post-traumatic pelvic hematomas, post radiation, or perforated hollow viscus including but not limited to: perforated appendicitis, bowel perforations due to foreign objects or malignancy, or perforated diverticulitis. Most of these conditions are usually found in the adult population. As per a recent literature search, there are no reported cases of a spontaneous retroperitoneal MRSA abscess in the pediatric population without risk factors.

## Introduction

Retroperitoneal abscesses are rare in infants and children [1]. They carry a high rate of morbidity and complications including, but not limited to, pneumonia potentially leading to respiratory failure, recurrent abscess, renal failure, cardiac disease, coloenteric fistula and osteomyelitis. The most frequent complication reported is pneumonia/respiratory failure with an associated mortality of 80% [2,3].

Potential sources of infection are the kidneys, ureters, pancreas, abdominal aorta and inferior vena cava. In adult populations, known causes of isolated retroperitoneal abscesses include osteomyelitis, seeding of post-traumatic pelvic hematomas, post-radiation, perforated appendicitis, perforated colon carcinoma or perforations due to a foreign object, diverticulitis, Crohn’s disease, cryptogenic and iatrogenic factors, acute cholecystitis and pancreatitis [3].

A retrospective summary of 41 children with retroperitoneal abscesses (anterior retroperitoneal abscesses, posterior retroperitoneal, retrofascial, and pelvic retroperitoneal abscesses) demonstrated 17% aerobic bacteria, 7% anaerobic and in 76% a combination of aerobic and anaerobic bacteria. Thirty-four percent of the cases showed polymicrobial infections [1]. The majority of the aerobic and anaerobic facultative isolates were *Escherichia coli* and *Staphylococcus aureus* while the majority of anaerobes were *Peptostreptococcus spp*, *Bacteroides spp*, *Prevotella spp* and *Clostridium perfringens* [1]. In the pediatric population, there are two case reports of patients being diagnosed with a retroperitoneal MRSA abscess, one patient with osteomyelitis and one with perforated appendicitis [2]. All of the cases reported with retroperitoneal abscess were secondary to other primary sources.

The literature reported incidence for retroperitoneal abscesses is highest in males between 30 to 60 years of age with a very limited number of cases reported in pediatric populations [4]. In the adult population, patients generally present with back pain and fever [5] and in these cases skin lesions were the presumed portals of entry for bacteria [6]. Patients may also have a history of anemia, hypoprothrombinemia, chills, abdominal or flank pain, nausea, vomiting, night sweats, and weight loss as concomitant factors [5,6].

Community-acquired methicillin-resistant *Staphylococcus aureus* (CA-MRSA) has shown an increase in incidence in both healthy pediatric and adult populations. In adults, a retrospective study (age range 17-65) of CA-MRSA between 2004 -2005 demonstrated that decreased hemoglobin (6.3%) and low serum prothrombin (52%) are associated with CA-MRSA infection [6]. There is no current data that extrapolates this to the pediatric population, but is something to consider in pediatric CA-MRSA infection. In CA-MRSA cases, blood cultures were only positive in 23% of cases and urine cultures were negative in all patients [6]. In 90% of cases there were predisposing or simultaneous clinical conditions such as ruptured appendix, trauma, previous surgery, remote infection, Crohn’s disease, splenectomy, immunodeficiency, osteomyelitis, diabetes, malignancy, steroid therapy, rupture of a hollow viscus and renal transplant [1]. As per a recent literature search, there are no reported cases of spontaneous retroperitoneal MRSA abscess in the pediatric population.

## Case presentation

An otherwise healthy two-year-old boy presented with an eight-day history of high-grade fevers up to 105F associated with vomiting, poor enteral intake, and abdominal pain. His mother reported that two weeks prior, the patient was playing with his family member who was sick with a cough and then he developed similar symptoms. The patient was treated with ibuprofen and his fever subsided, but then complained of a burning sensation with urination. He was prescribed amoxicillin for urinary tract infection and completed three days of a seven-day course. Two days later, the fever and cough returned prompting a visit to the emergency room where the patient was found to be influenza A positive and subsequently sent home with supportive management. Following discharge, he experienced refractory daily fevers between 101F and 105F, intermittent vomiting and coughing with post-tussive emesis. The patient was subsequently admitted to the hospital due to dehydration and abdominal pain.

During hospitalization the patient was febrile to 103F and was tachycardic, averaging 140-160 BPM. Laboratory results were remarkable for leukocytosis of 23,500 cells per microliter with bandemia of 29,000 cells per microliter, elevated c-reactive protein of 5.32mg/dL and procalcitonin that was 4.8ng/mL. Chest x-ray, blood-urine cultures, and urinalysis were all negative.

**Figure 1:**
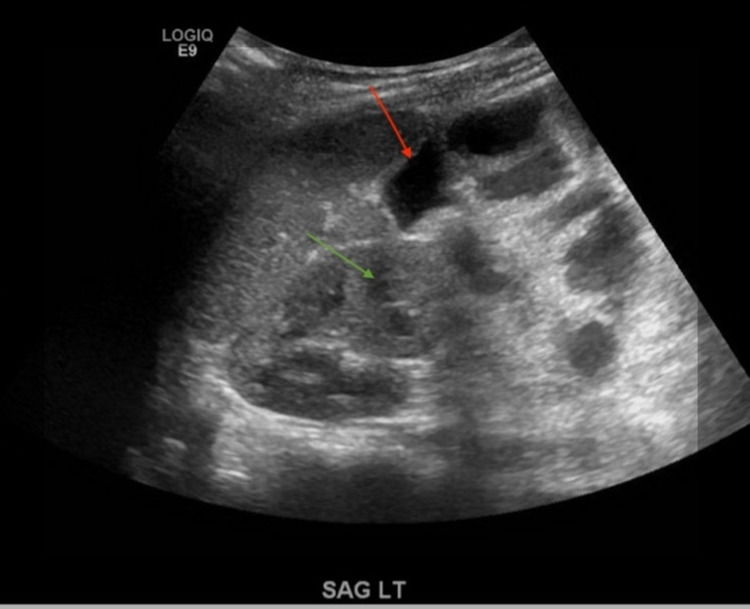
Abdominal ultrasound of right kidney with hydronephrosis(green arrow) and prominent bowel loops(red arrow).

**Figure 2:**
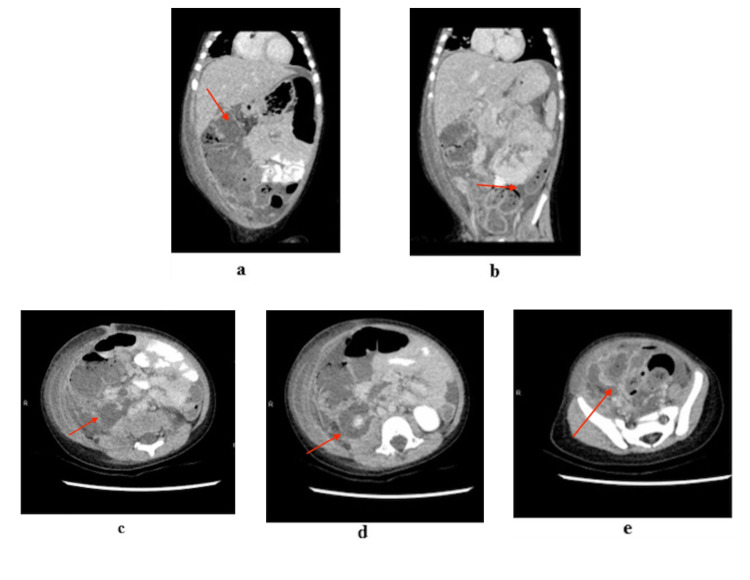
CT Abdomen and pelvis with PO contrast: (a) Hypodense fluid throughout the large bowel; dilated ascending colon with pneumatosis intestinalis. (b) large volume ascites with patchy peritoneal enhancement towards the abdominopelvic junction. (c) and (d) moderate volume right-sided perinephric fluid (e) Oblong rim-enhancing collection within the abdominopelvic junction

An ultrasound study of the abdomen was unable to localize any pathologies, including pyelonephritis (Figure 1). CT of the abdomen and pelvis showed large volume ascites with patchy peritoneal enhancement towards the abdominopelvic junction. Hypodense fluid was seen throughout the large bowel; there was a dilated ascending colon with pneumatosis intestinalis, possible bowel ischemia. An oblong rim-enhancing collection was noted within the abdominopelvic junction. There was also moderate volume right-sided perinephric fluid (Figure 2).

The patient was started on intravenous vancomycin and piperacillin-tazobactam and taken to the operating room for an exploratory laparotomy. Upon entering the peritoneum, clear ascites was encountered. The ascending colon was found to be intact with no pneumatosis or perforation. The appendix was noted to be hyperemic with no perforation; therefore, an appendectomy was performed. An abscess was noted tracking from the retroperitoneum with inflamed Gerota’s fascia and retroperitoneal portion of the duodenum. No perforation or pneumatosis was noted in the duodenum. A biopsy of Gerota’s fascia and culture from the abscess was sent to the laboratory. The abdomen was washed out with warm crystalloid solution. A temporary abdominal closure was performed and the patient was taken to pediatric ICU intubated and on a mechanical ventilator. On post-operative day four, the patient was taken back to operating room for a second look, further wash out, and abdominal closure.

Cultures from abscess grew methicillin-resistant *Staphylococcus aureus*. Repeat urinalysis and blood cultures showed no growth. Ultrasound of the hips showed no indicators of septic hip. Further workup with voiding cystourethrogram showed no evidence of vesicoureteral reflux during the filling or voiding phase. Parenteral nutrition was initiated. Patient was extubated on post-operative day five. Leukocytosis trended down. A peripherally inserted central catheter line was placed and the patient was discharged to a rehabilitation facility on hospital day 16 with ceftaroline and metronidazole.

## Discussion

The retroperitoneum is described as a potential cavity with specific boundaries: between the peritoneum and transversalis fascia lining the posterior abdominal cavity, extending laterally to the edges of the quadratus lumborum muscles, the diaphragms superiorly, and the pelvis inferiorly. It includes an anterior retroperitoneal space and a posterior space, the latter being further divided into the perinephric spaces that extend across the midline. The lateral fossa on each side is further divided by the renal fascia which separates into an anterior and posterior layer that surrounds the kidneys and passes medially in front of and behind the great vessels. The renal fascia compartment is open inferiorly but closed superiorly [5]. The kidneys, ureters, the duodenum, ascending and descending colon, cecum, abdominal aorta, and pancreas are all located within the retroperitoneal space.

In the adult population, an average of 12.7 days is required to complete the diagnosis of retroperitoneal abscess [3]. Retroperitoneal abscess is a condition that is mainly diagnosed with imaging. Computed tomography (CT) is valuable for the diagnosis of abdominal abscesses, especially visceral and retroperitoneal abscesses [2,4,6]. Plain abdominal x-rays may be useful for initially evaluating abdominal pain due to abscesses. Ultrasound is also a useful tool to detect intra-abdominal abscesses, particularly to spare radiation in the pediatric population. Ultrasonography is more specific than plain abdominal x-rays for the diagnosis of appendiceal abscesses. When a focus of infection is challenging to identify, granulocyte scintigraphy can be utilized [2]. Plain abdominal films will show an abnormality in 38% to 90% of patients such as loss of renal outline, scoliosis or a soft tissue mass. Plain chest radiography is able to show elevation of the ipsilateral diaphragm, pleural effusion or basilar atelectasis caused by intra-abdominal pathology. Intravenous pyelography can be used to show orthotopic location of the kidneys or deviation of ureters. Barium contrast studies can show displacement of the viscera, extravasation of contrast, and in rare cases a fistulous tract [4].

The approach to treatment of intra-abdominal abscesses includes drainage, treatment of the primary source, and administration of antimicrobials [1]. Abscesses can be drained by open surgery or percutaneous drainage under ultrasound or CT guidance [6]. The surgical approach should be carefully considered in concordance with available imaging. Transperitoneal drainage should be avoided if possible as one case series of 50 patients showed a 67% failure rate [3]. The Centers for Disease Control and Prevention recommends vancomycin as monotherapy or combined with trimethoprim-sulfamethoxazole, gentamicin, or clindamycin as first-line antibiotic therapy for severe infection caused by CA-MRSA [6]. One study shows a 90% success rate with percutaneous drainage of intra-abdominal abscesses and long-term antibiotic therapy, but this study includes few retroperitoneal abscesses. It is still most reasonable to treat retroperitoneal abscesses with percutaneous drainage and antibiotics. Open surgical drainage should be considered if percutaneous drainage is inadequate [3].

The patient in this case report presented with acute abdominal pain with radiological findings concerning for bowel ischemia of the ascending colon with a retroperitoneal abscess. Differential diagnosis at the time of presentation included potential sources of infection from kidneys, ureters, bowel, or bone. Secondary causes of retroperitoneal abscess include osteomyelitis, seeding of post-traumatic pelvic hematomas, post-radiation, perforated appendicitis, perforated hollow viscus due to carcinoma or foreign object, diverticulitis, Crohn’s disease, cholecystitis, pancreatitis, cryptogenic and iatrogenic factors. Urinalysis, voiding cystourethrogram, ultrasound of the hip did not reveal the primary cause of the abscess. None of the aforementioned sources had a localized infection. This patient was treated with antibiotics and surgical management because of pneumatosis on CT scan. Cultures obtained from the abscess grew MRSA. The patient’s clinical status and hemodynamics improved after undergoing surgical intervention, admission to the intensive care unit, parenteral nutrition support, and long-term antibiotics. Patient was discharged on day 16 of hospitalization to a rehabilitation facility. He was seen within two weeks in the office, feeling well without complaints.

## Conclusions

We report a spontaneous retroperitoneal abscess with methicillin-resistant *Staphylococcus aureus* bacteria in a pediatric patient. Secondary causes of retroperitoneal abscesses in pediatric patients include osteomyelitis, seeding of post-traumatic pelvic hematomas, post-radiation, perforated appendicitis, perforated hollow viscus due to foreign object, Crohn’s disease, cryptogenic and iatrogenic factors. Identifying the source of infection should be done on initial presentation. Appropriate antibiotics choice and percutaneous drainage are the main forms of treatment. Open surgical drainage of abscesses should be considered if percutaneous drainage is inadequate. This is a unique case not previously described in the literature. Spontaneous retroperitoneal abscesses are a rare disease that can be considered as a diagnosis if all secondary causes have been ruled out and no source can be identified. 
